# Supplementary material for: Influenza A Virus Infection Reactivates Human Endogenous Retroviruses Associated with Modulation of Antiviral Immunity
Source: Viruses. 2022 Jul 21;14(7):1591. doi: 10.3390/v14071591 (PMC9320126; doi:10.3390/v14071591)
Supplement: Supplementary file 1 [file viruses-14-01591-s001.zip › MotifAnalysis.zip!/MotifOutput_PR8M/homerResults/motif10.similar.html]

motif10

## Information for motif10

C
G
T
A
A
C
G
T
C
G
T
A
A
G
T
C
C
G
T
A
A
G
T
C
A
G
T
C
C
G
T
A
C
G
T
A
A
C
G
T
A
G
T
C
A
C
T
G
  
Reverse Opposite:  

A
G
T
C
A
C
T
G
C
G
T
A
A
C
G
T
A
C
G
T
A
C
T
G
A
C
T
G
A
C
G
T
A
C
T
G
A
C
G
T
C
G
T
A
A
C
G
T
  

|  |  |
| --- | --- |
| p-value: | 1e-14 |
| log p-value: | -3.242e+01 |
| Information Content per bp: | 1.530 |
| Number of Target Sequences with motif | 7.0 |
| Percentage of Target Sequences with motif | 30.43% |
| Number of Background Sequences with motif | 76.7 |
| Percentage of Background Sequences with motif | 0.17% |
| Average Position of motif in Targets | 118.4 +/- 47.1bp |
| Average Position of motif in Background | 87.8 +/- 93.2bp |
| Strand Bias (log2 ratio + to - strand density) | 10.0 |
| Multiplicity (# of sites on avg that occur together) | 1.43 |
| Motif File: | file (matrix) reverse opposite |

### Similar de novo motifs found

|  |  |  |  |  |  |  |  |
| --- | --- | --- | --- | --- | --- | --- | --- |
| Rank | Match Score | Redundant Motif | P-value | log P-value | % of Targets | % of Background | Motif file |
| 1 | 0.815 | A G T C C G T A G T A C A G T C C T G A C T G A A C G T T G A C | 1e-11 | -26.081803 | 34.78% | 0.76% | motif file (matrix) |
| 2 | 0.913 | C G T A A C G T C G T A A G T C C G T A A G T C A G T C C G T A C G T A A C G T | 1e-10 | -23.420865 | 34.78% | 1.06% | motif file (matrix) |
